# Rapid identification and quantitation of single plant seed allergen using paper-based microfluidics

**DOI:** 10.1371/journal.pone.0266775

**Published:** 2022-12-12

**Authors:** Xiaodong Sun, Yongxin Liu, Bing Niu, Qin Chen, Xueen Fang

**Affiliations:** 1 School of Medicine, Shanghai University, Shanghai, P.R. China; 2 Shanghai Key Laboratory of Bio-Energy Crops, School of Life Sciences, Shanghai University, Shanghai, P.R. China; 3 Department of Chemistry and Institutes of Biomedical Sciences, Fudan University, Shanghai, P.R. China; Cairo University, EGYPT

## Abstract

Nucleic acid amplification is a sensitive and powerful tool for allergen detection. However, it is limited due to the relatively cumbersome methods required to extract nucleic acids from single plant seed allergen (e.g. peanut and soybean). In view of this, an approach of extracting nucleic acid with untreated glass-fiber paper (paper-based microfluidics) was applied for nucleic acid capture and purification from plant seed allergen and commercial products. After cut by hollow cylindrical cutter, a certain size the paper chip it used to absorb DNA. And this paper-based microfluidics with DNA was directly applied for amplification by loop-mediated isothermal amplification (LAMP). To evaluate the adsorption performance of paper chip to DNA, CTAB and SDS method were used as comparisons. From amplification results, the established technique has good specificity, high repeatability (C.V. values are 4.41% and 6.17% for peanut and soybean) and favorable sensitivity (7.39 ng/μL or peanut and 6.6 ng/μL for soybean), and successfully used for commercial products (2 kinds of candy and 2 kinds of cakes containing peanut, and 2 kinds of drinks, candy and 2 kinds of biscuits containing soybean). This speed and flexible detection method makes it suit for applications in point-of-care (POC) detection at different scenario, such as custom house and import port.

## 1. Introduction

In recent years, increasing attention has been paid to the point-of-care (POC) detection of nucleic acids *in vitro*. This method facilitates rapid, sensitive, and user-friendly analysis in non-laboratory and resource-limited environments (Dai et al., 2020). Compared with traditional laboratory methods, the POC detection of nucleic acids can realize the rapid detection of “injection and output” without the need for large and expensive equipment [1, 2]. Paper-based equipment is widely used in POC diagnosis, food safety analysis, pathogen detection, and environmental detection [3–7]. In recent years, significant progress has been made in paper-based microfluidic detection methods [8–10]. Glass fiber paper has a capillary structure, so that the liquid flows spontaneously owing to capillary action, without an external pump to manipulate the fluid; additionally, glass fiber paper is biochemical/chemically inert [11, 12]. These properties make glass fiber paper a good candidate for the matrix of paper-based microfluidics. After modifying the surface of fiberglass paper, Adiguzel et al. (2014) detected visually the yeast cells fixed on the surface using Gram staining; additionally, DNA was detected by complementary DNA sequences on the fiber paper surface and visually detected by YOYO-1 [13]. Fang et al. used graphene oxide (GO)-modified glass fiber paper and ssDNA with fluorescent groups for the rapid and low-cost detection of biological macromolecules [14].

Nucleic acid extraction is the first and most critical step for POC detection. However, the traditional methods of extracting DNA from plant tissues are complex and time-consuming. An example of such methods is the cetrimonium bromide (CTAB) precipitation method, which is widely used in the extraction of nucleic acids from plant samples [15–17]. The CTAB extraction process mainly includes mechanical grinding, chemical reagent cell lysis, temperature-assisted incubation, and organic-phase nucleic acid extraction. In recent years, other extraction methods, such as silica gel column extraction, salt extraction, and micro- and nanomagnetic particle extraction methods, have been developed to solve these problems [18–20]. However, these extraction methods are still slightly cumbersome, time-consuming, and labor-intensive [21]. Therefore, it is important to develop a rapid nucleic acid extraction method. For example, Shi et al. combined a glass fiber filter (a silicon-based mineral material) in a commercial kit with filter paper for the high-throughput extraction of plant DNA, and the extracted DNA could be used as a PCR template [22].

Peanuts and soybeans are important ingredients in the daily diet and have rich nutritional value. Peanuts and soybeans contain a variety of trace elements that promote the growth and development of the human body. However, peanuts and soybeans are also food allergens that can cause a sudden increase in the incidence of allergic diseases [23]. Research has reported that peanut allergies account for 10–47% of the total number of allergies, and soybean allergies account for 25% of the total number of food allergies [24]. As two of the most serious food allergens, peanuts and soybeans mainly cause allergies manifesting in the skin, digestive tract, respiratory tract, and with symptoms such as swollen lips, itching, angioedema, rhinoconjunctivitis, asthma, nausea, and vomiting; sometimes, these allergens can cause systemic or even life-threatening reactions, such as anaphylactic shock [25]. However, to date, there is no effective treatment for food allergies [26]. People with food allergies must eat only a specific diet to prevent the occurrence of allergic reactions; therefore, it is very important to detect trace allergenic ingredients in food. As a real-time testing method, POC has many advantages, as it has a few processing steps, a short detection cycle, and low inspection costs, while it is also fast and simple, which means that it can be used as a supplementary method for regular detection methods. Loop-mediated isothermal amplification (LAMP) is widely used in the DNA detection of peanuts, soybeans, shrimp, fish, and other allergens, and exhibits characteristics ideal for POC applications, as it is less demanding in terms of the required instrumentation [27–30].

Herein, a simple, equipment-free method that can be used to extract and purify DNA from commercial products were described. The method was combined with paper microfluidics (extraction part) and LAMP (detection part) and was used for the amplification of plant seed allergens (peanut gene *Ara h 6* and soybean gene *Gly m Bd 28 K*). After evaluating the specificity, repeatability, and sensitivity of the method, commercially available products containing peanuts and soybeans were used to successfully extract and detect the DNA of these allergens. The methods described in this study have the potential to be effectively applied to remote areas and POC detection.

## 2. Materials and methods

### 2.1 Reagents, samples and instruments

Pearl red-skin peanuts were purchased from Wal-Mart supermarket in different city (Hangzhou, Qingdao, Beijing, Suqian, and Heishan, China), white jade peanuts, selenium-enriched black peanuts were obtained from supermarket in Heishan (China). Yellow soybeans (purchased from Wal-Mart supermarket in Linyi, Nanjing, Hangzhou, Jiamusi, and Heishan), black soybeans, and green soybeans (both purchased from Wal-Mart supermarket in Heishan) were used as soybean samples. Sesame seeds, pistachios, walnuts, almonds, and commercial products were purchased from the Shanghai Wal-Mart supermarket. Bst DNA polymerase, a 10× isothermal amplification buffer, a dNTP mixture, and an MgSO_4_ solution were purchased from Sangon Biotech (Shanghai, China). DNA markers were purchased from Shanghai Jierui Biological Engineering Co. Ltd. Sample lysis and washing buffers were purchased from Shanghai Suchuang Diagnostic Products Co., Ltd. Glass fiber paper (BT50) was purchased from Shanghai Jinbiao Technology Co., Ltd (Shanghai, China). The instruments used in this experiment were a real-time PCR instrument (Bio-Rad Biotechnology Co., Ltd., USA), an electrophoresis tank, an electrophoresis instrument, a Tanon 3500 automatic digital gel imaging system (Shanghai Tianneng Technology Co., Ltd., Shanghai, China), a Lambda 650 solid ultraviolet spectroscopy photometer, a trace ultraviolet spectrophotometer, and an Olympus IX73 fluorescence microscope (Olympus, Japan).

### 2.2 Paper chip production and nucleic acid extraction

A hollow cylindrical cutter was used to cut the fiberglass paper into round extraction chips (3 mm diameter) and washing chips (30 mm diameter). Then, the sample was ground into a powder, 50 mg of which was weighed, placed in a 300–400 μL lysis buffer solution, fully shaken, and mixed into a homogenate that was incubated for 10–15 min for cell lysis to release nucleic acid. Then, the homogenate was loaded onto the extraction chip, the chip was placed on the washing chip with tweezers, and the washing buffer was added to wash the homogenate twice and remove the polysaccharides and proteins.

### 2.3 Adsorption performance of paper chip to DNA

Peanut DNA was used to test the adsorption capacity of the paper chips used in this method for nucleic acids. First, peanut DNA and negative samples (ultrapure water without DNase/RNase) were mixed with DNA dye (1:2500 dilution) and incubated in the dark for 5 min. Then, 10 μL was added to the surface of the paper chip, and the fluorescence signals of the paper chip before and after washing were observed by fluorescence microscopy to verify the DNA adsorption capacity of the paper chip.

### 2.4 LAMP detection and agarose gel electrophoresis

The total volume of the LAMP amplification system used in the experiment was 25 μL, including 7.5 μL of the LAMP working solution (1× isothermal amplification buffer, 6 mM MgSO_4_, and 1.4 mM dNTP mixture), 1.1 μL of the primer working solution (FIP/BIP: 1.6 μM, FL/BL: 0.8 μM, F3/B3: 0.2 μM; if there is no loop primer, ultrapure water can be used instead), 1 μL of Bst polymerase, and 1 μL of fluorescent dye (SYTO 9). The rest of the volume was made up by water. The paper chip with nucleic acid was extracted into the configured LAMP amplification system, mixed evenly, and centrifuged until no bubbles were present in the system.

The primers used for the peanut allergen encoding gene *Ara h 6* (NCBI GenBank, EF609643.1) and soybean allergen encoding gene *Gly m Bd 28 K* (NCBI GenBank, AF240005.1) were the same as those used in a previous report [31].

Next, we performed agarose gel electrophoresis of the amplified product. The LAMP reaction product (5 μL) was mixed with 1 μL of 6 × loading buffer and then added to a 1% agarose gel sample hole and electrophoresed in a 1 × TAE buffer. The electrophoretic voltage was set at 85 V and electrophoresis lasted 45 min. Then, the results were analyzed using a gel imaging system.

### 2.5 Calculation

The data are expressed as the mean ± SE with three time experiments. Repeatability data were analyzed to determine statistical significance using SPSS (version 13.0, USA).

## 3. Results and discussion

### 3.1 Process of paper microfluidic chip for rapid extraction of nucleic acids

**Fig 1:**
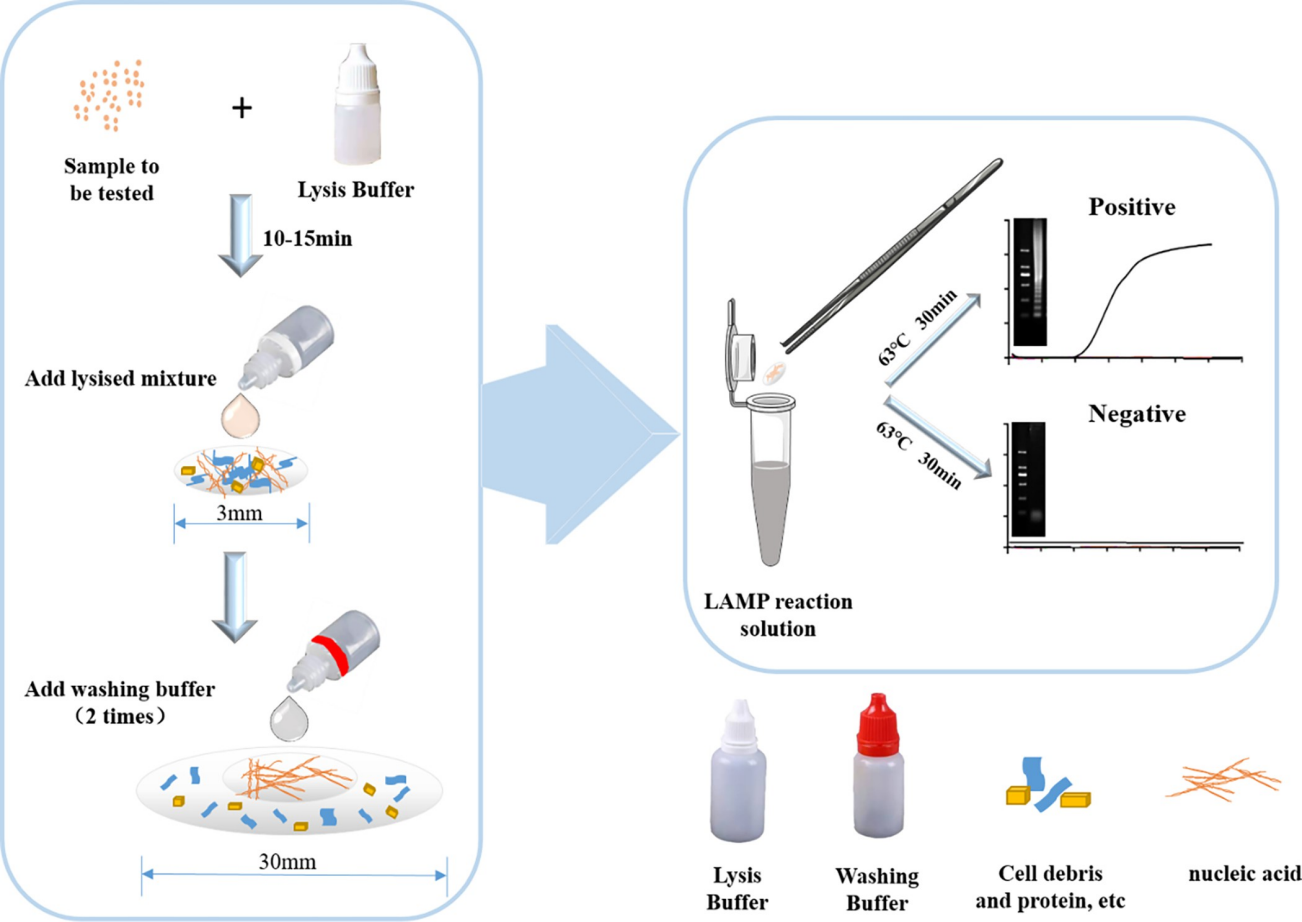
Process of LAMP method for nucleic acid detection extracted by paper-based microfluidics.

The design and operational process of the LAMP detection method based on nucleic acid extraction on a paper chip are shown in **Fig 1**. This process consisted of two stages. First, a nucleic acid extraction process based on a paper chip was introduced. The peanut or soybean sample ground into powder form was added into a drop bottle containing lysis buffer, and the mixture was shaken sufficiently to mix evenly. The mixture was allowed to stand for 10–15 min for full lysis and then it was added to the paper chip to adsorb nucleic acids. The paper chip was then washed with washing buffer to remove potential LAMP amplification inhibitors from the pyrolyzed sample. The extraction chip with the extracted nucleic acid was then placed in the LAMP amplification reaction solution, and the DNA extracted on the paper chip was used as the template for the LAMP reaction. Therefore, the entire nucleic acid extraction and detection process is simple and convenient and can be completed within 1 h at most. To prove the superiority of combination of paper based microfluidics with LAMP, a primary experiment of the extraction DNA of peanut and soybean for LAMP detection was performed and a comparison of studies on paper-based microfluidics and other topics is presented in **S1 File**.

### 3.2 Adsorption and extraction performance of paper microfluidic chip to DNA

**Fig 2:**
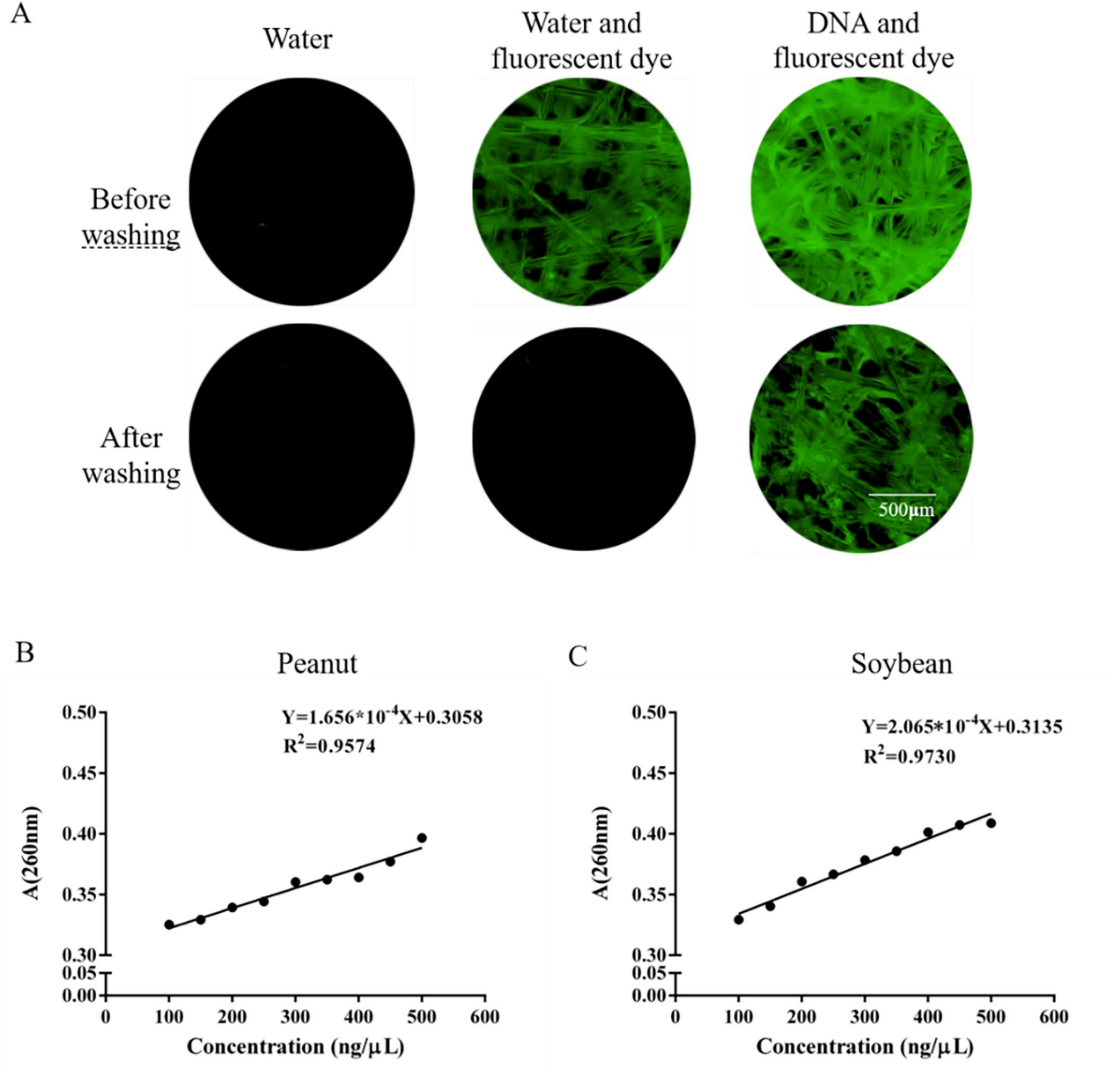
Adsorption of DNA and H_2_O on paper-based microfluidics. Notes: A, fluorescence of paper chip; B and C standard line of different concentration of DNA.

To test the adsorption performance of the glass fiber paper chip in this method, pure peanut DNA was extracted in advance for verification. After adding water and the fluorescent dye premix, DNA and the fluorescent dye premix were added to the paper chip for incubation and washing, as is shown in **Fig 2A**. It was observed that in the washed chips, only the premix with DNA and fluorescent dyes still had strong fluorescence under the fluorescence microscope, while the paper chips containing other samples hardly exhibited fluorescence after washing, which suggests that the paper chip had a strong adsorption effect on DNA. Many previous studies have shown that DNA can be purified from complex biological samples by the adsorption of silica on DNA under conditions of chaotropic salts [32]. The main component of glass fiber is silica, which has electrostatic interactions with the negatively charged phosphate groups of nucleic acids. In addition, as a physical network, glass fiber paper can capture and adsorb nucleic acids in low-salt and low-pH buffers.

After washing, the binding between the glass fiber and nucleic acids was not affected. Moreover, owing to the natural capillary force of the paper, the washing solution removed the amplification inhibitors, such as proteins and polysaccharides, from the paper chip during the washing process to separate the nucleic acid from these amplification inhibitors. As a fast, simple, and flexible method, paper-based microfluidic chips can achieve nucleic acid extraction in a short time.

Simultaneously, different concentrations of peanut DNA and soybean DNA (100, 150, 200, 250, 300, 350, 400, 450, and 500 ng/μL) were added to the paper chip, and the different DNA concentrations on the paper chip and its absorbance at 260 nm were measured using a Lambda 650 solid ultraviolet spectrophotometer. The results shown in **Fig 2B and 2C** suggest that after DNA samples with different concentrations were added to the paper chip, a good linear relationship between the A260 value and the concentration was observed (peanut DNA: Y = 1.656 × 10^-4^X + 0.3058; soybean DNA: Y = 2.065 × 10^-4^X + 0.3135), and the R^2^ values were 0.9574 (peanut) and 0.9730 (soybean).

### 3.3 Comparison of paper chip nucleic acid extraction method with CTAB and SDS extraction methods

During a nucleic acid test (NAT), nucleic acid extraction is a critical step. Among the currently available extraction methods of peanut and soybean DNA, CTAB and sodium dodecylbenzene sulfonate (SDS) are the most traditional ones. To verify the effect of nucleic acid extraction methods based on paper chips, different extraction methods were utilized to extract the same amount of peanut and soybean samples, and the extraction effects (extraction amount, purity) and extraction time of different methods were determined. The results are presented in **Table 1**. The results show that although the CTAB and SDS methods are slightly more efficient than paper chip nucleic acid extraction methods, these extraction methods not only require heating incubation, high-speed centrifugation, multiple washing, and other steps, but also require the use of large-scale equipment, such as centrifuges and water bath pots or the use of chloroform, isopropanol, etc., during the extraction process; these extra steps result in the entire extraction process being time-consuming and laborious. Toxic reagents will harm the human body after long-term use.

**Table 1:** Comparison of paper chip nucleic acid extraction method and other methods.

Sample	Extraction method	DNA extraction volume (μg)	A260/A280	Extraction time	Heated or not	Toxic or not
Peanut	Paper chip method	245.17±23.05	1.09±0.02	About 20 minutes	No	No
	CTAB method	421.48±1.04	1.84±0.01	About 105 minutes	Yes	Yes
	SDS method	353.70±0.82	1.95±0.01	About 110 minutes	Yes	Yes
Soybean	Paper chip method	448.42±12.39	1.09±0.04	About 20 minutes	No	No
	CTAB method	735.86±0.72	1.81±0.02	About 105 minutes	Yes	Yes
	SDS method	738.96±0.54	1.78±0.01	About 110 minutes	Yes	Yes

Note: The initial extraction amount of peanuts and soybeans in the three different extraction methods is 200 mg.

Compared to the aforementioned methods, the paper chip extraction method described herein is simple and inexpensive, while the required materials are easy to obtain. The paper chip extraction method can be applied quickly and conveniently in any laboratory or POC system. The entire extraction process of this method is simple and fast; it only consists of two steps, cracking and washing, and can be completed in 20 min, without the assistance of a centrifuge or other equipment. At the same time, toxic reagents are not required during the extraction process, while the fiberglass paper used is environmentally friendly and can be directly incinerated after use, which greatly reduces biological pollution.

### 3.4 Specificity of paper microfluidics for the nucleic acids extraction/detection for peanut and soybean allergens

**Fig 3:**
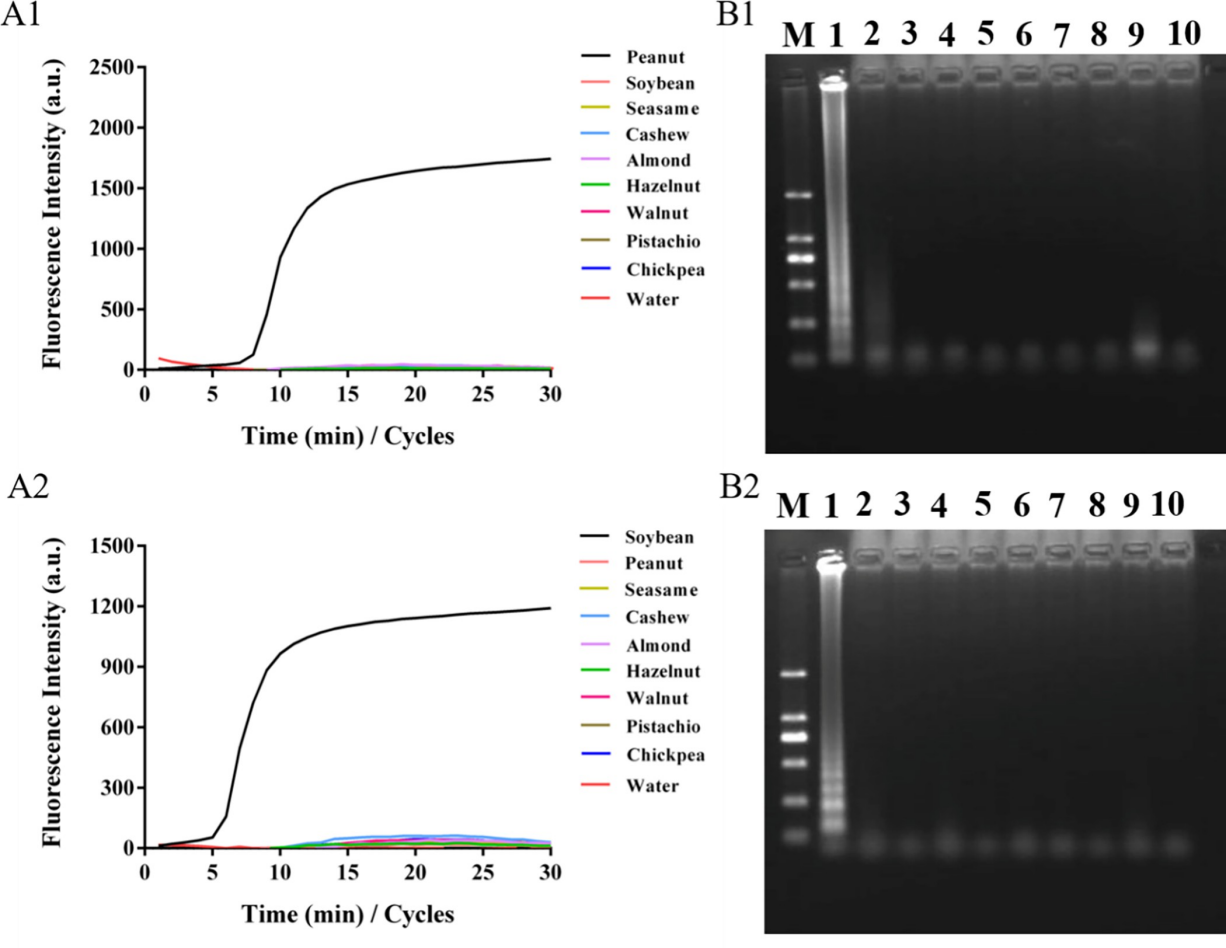
Specificity of LAMP detection with paper-based microfluidics extraction. Notes: A1 & A2, LAMP amplification of peanut and soybean; B1 & B2, corresponding ladder electrophoresis bands of peanut and soybean.

**Fig 4:**
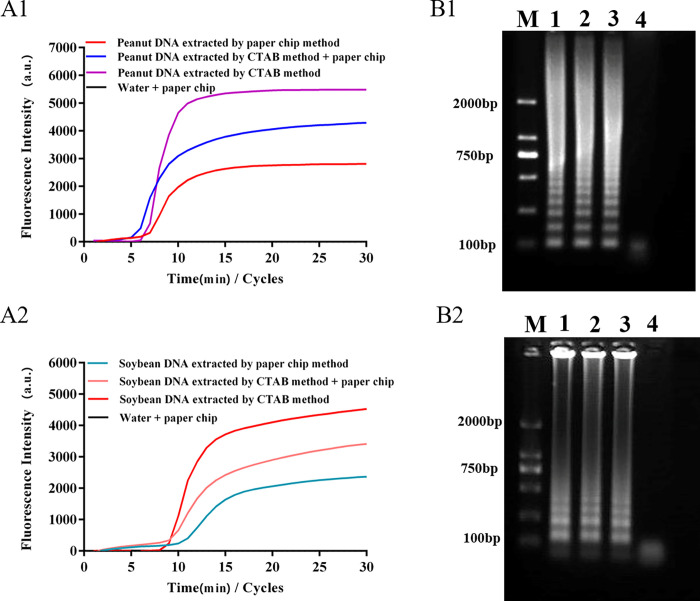
LAMP detection and electrophoresis verification based on the extraction DNA by paper-based microfluidics. Notes: A1 & A2, LAMP amplification curves of peanut and soybean; B1 & B2, corresponding ladder electrophoresis bands of peanut and soybean.

Peanut and soybean DNA extracted by the paper chip method combined with the LAMP method was used for the specific detection of peanut and soybean allergens. When testing for peanut or soybean allergens, the peanut, soybean, sesame, cashew, walnut, almond, pistachio, hazelnut, and chickpea samples were initially ground into powder. Then, nucleic acid was extracted from the nine samples using a paper chip, and the paper chip with the extracted DNA was placed into the LAMP amplification system for specific detection. **Fig 3A** shows the specific test results for peanut samples. The soybean, sesame, cashew nut, walnut, almond, pistachio, hazelnut, and chickpea samples were selected as negative samples and ultrapure water was used as a blank sample to prove the specificity of our detection method. The results showed that only the peanut samples exhibited an S-shaped amplification curve with a Ct value of 8, while the other negative and blank samples did not show amplification, indicating that the method had good specificity. Agarose gel electrophoresis of the amplified products of LAMP showed that only the ladder electrophoresis bands appeared in the peanut samples, while the other negative samples and blank samples exhibited no ladder-like electrophoresis bands, which further verified the results of the LAMP reaction. The S-type amplification curve of the soybean allergen and its corresponding ladder electrophoresis bands (**Fig 4A1 and 4B1**) were obtained by the same method, with a Ct value of 6. No amplification curve or ladder band was observed in the other negative and blank samples. These results indicate that the LAMP detection method based on paper chip nucleic acid extraction can be used for the specific detection of peanut and soybean allergens.

### 3.5 Sensitivity of paper microfluidics for the nucleic acids extraction/detection for peanut and soybean allergens

**Fig 5:**
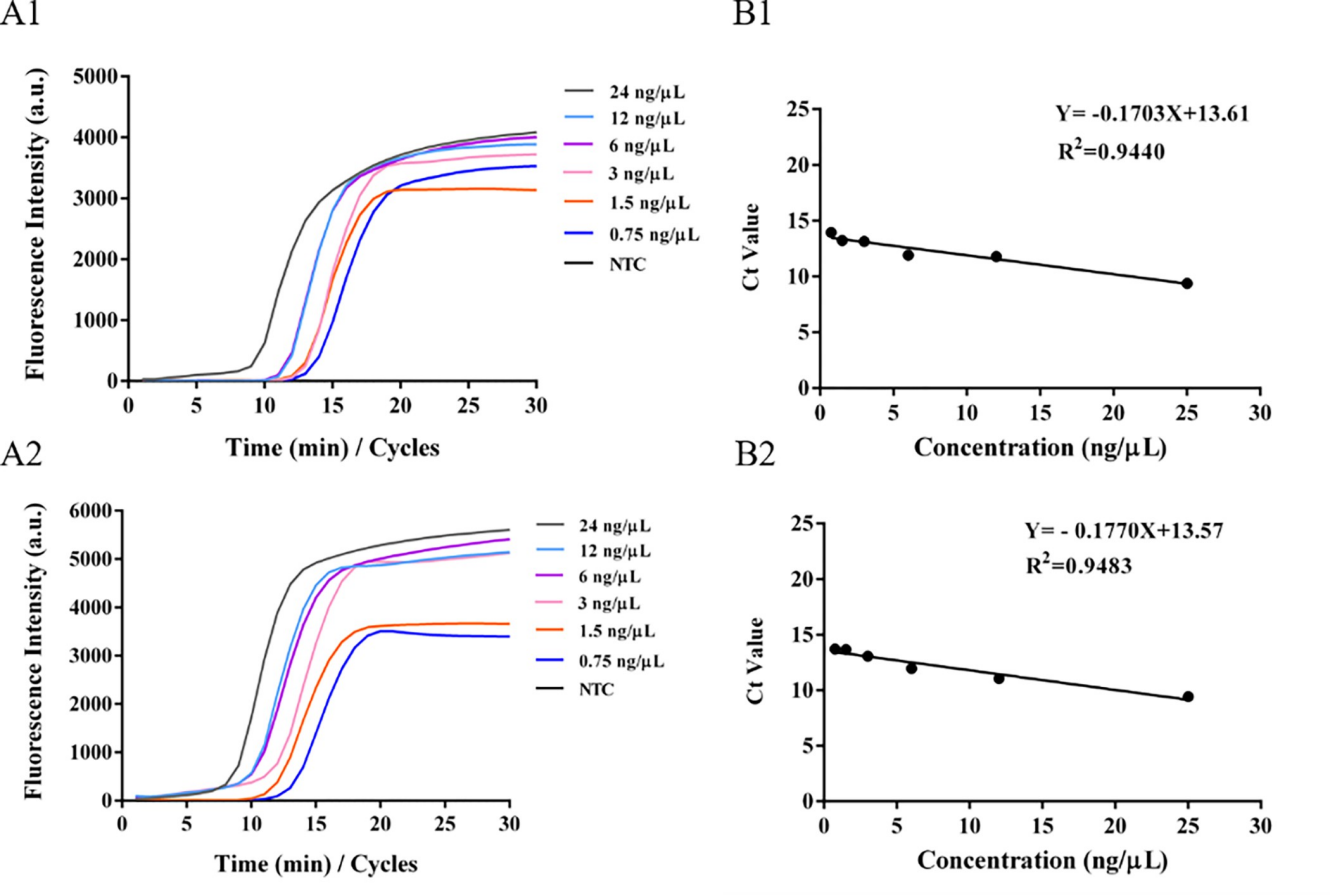
LAMP detection of different concentrations of DNA on paper-based microfluidics. Notes: A1 & A2, amplification curves of peanut and soybean DNA with different concentrations; B1 & B2, linear relationship between peanut and soybean DNA concentrations and Ct value.

To verify the lowest detection limit of the lamp method for peanut and soybean DNA extracted using a paper chip, different concentrations of peanut and soybean DNA (0, 0.75, 1.5, 3, 6, 12, and 24 ng/μL) were selected. The samples were loaded onto a paper chip and placed in LAMP amplification buffer for amplification. A linear curve was established based on the amplified Ct value and concentration. **Fig 5A1 and 5A2** show the fluorescence amplification curves of peanut and soybean DNA at different concentrations on the paper chip, showing a good amplification effect. The results shown in **Fig 5B1 and 5B2** suggest that there was a good linear relationship between different peanut or soybean DNA concentrations and their amplified Ct values (peanut DNA: Y = -0.1703X + 13.61; soybean DNA: Y = -0.1770X + 13.57), with R^2^ values of 0.9440 and 0.9483, respectively. These results show the lowest detection limit of the LAMP method for DNA extracted by the paper chip method according to the amplified Ct value of peanut or soybean nucleic acid.

**Fig 6:**
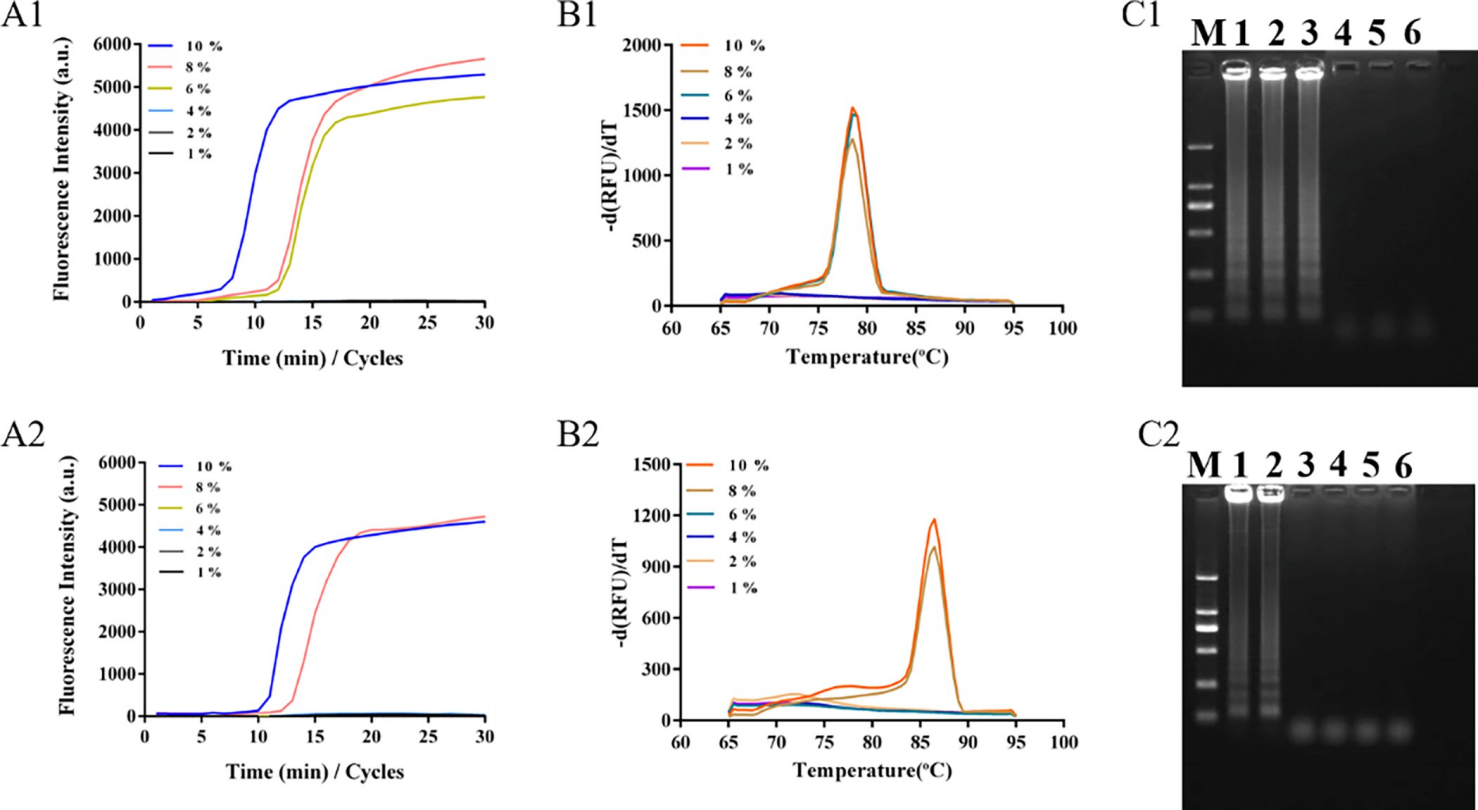
Sensitivity of LAMP detection with paper-based microfluidics extraction. Notes: A1, B1 & C1, the fluorescence amplification results, melting curve results, and agarose gel electrophoresis results of different peanut diluted samples; A2, B2 & C2, the fluorescence amplification results, melting curve results, and agarose gel electrophoresis results of different soybean diluted samples.

Then, the peanut and soybean samples were diluted to different concentrations (10%, 8%, 6%, 4%, 2%, and 1%) and subjected to paper chip nucleic acid extraction. The paper chip with the extracted nucleic acid was detected using the LAMP amplification system, and the lowest detection limit was quantified according to the linear curve. The results are shown in **Fig 6**. **Fig 6A1–6C1** show the fluorescence amplification results, melting curve results, and agarose gel electrophoresis results of different diluted peanut samples. The results showed that 10%, 8%, and 6% of the diluted peanut samples had amplification curves, and the Tm value in the melting curve was highly consistent, which indicated that the method could extract and detect 6% of the diluted peanut samples. Similarly, we obtained results for different concentrations of diluted samples (**Fig 6A2–6C2**). The results showed that the method could extract and detect 8% of the diluted soybean samples. In addition, according to the linear equation and amplified Ct value, the concentration was calculated to be approximately 7.39 ng/μL and 6.6 ng/μL, respectively.

### 3.6 Repeatability of paper microfluidics for the nucleic acids extraction/detection for peanut and soybean allergens

**Fig 7:**
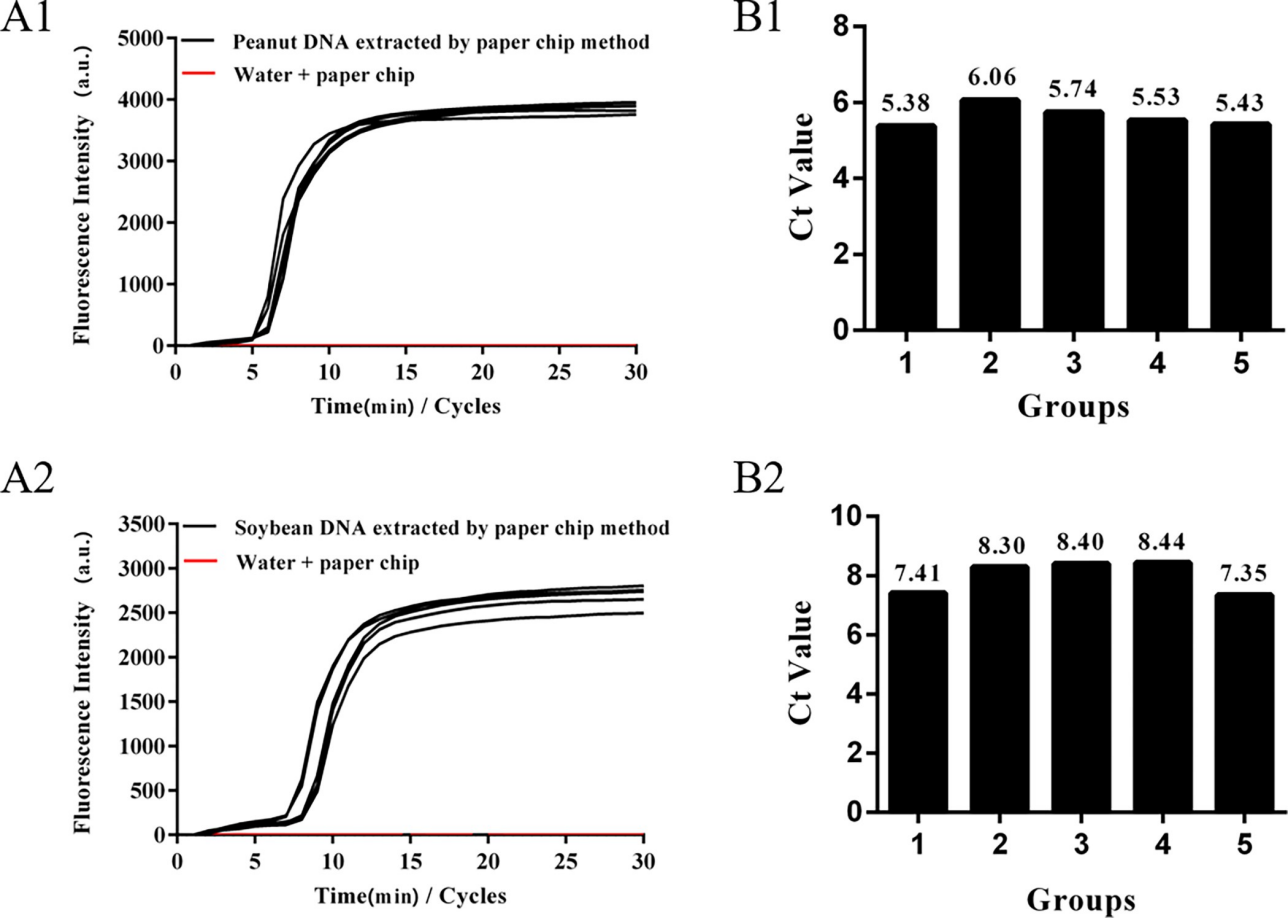
Repeatability of LAMP detection with paper-based microfluidics extraction. Notes: A1 & A2, the amplification results of peanut and soybean with 5 repeats; B1 & B2, Ct value of peanut and soybean with 5 repeats.

To verify the repeatability of the LAMP detection method for peanut and soybean allergens extracted by the paper chip method, the peanut and soybean powder sample analysis was repeated five times, the Ct values were recorded, and the corresponding standard deviation (SD) and coefficient of variation (CV) were calculated. The results are shown in **Fig 7**. When the paper chip nucleic acid extraction and LAMP methods were used to detect peanut (**Fig 7A1**) and soybean allergens (**Fig 7A2**), the amplified Ct values were stable, with SD values of 0.248 and 0.492, and CV values of 4.41% and 6.17%, respectively, indicating that the method had good repeatability for peanut and soybean allergen detection.

### 3.7 Applicability of LAMP detection method for peanut and soybean allergens extracted from paper chip

**Fig 8:**
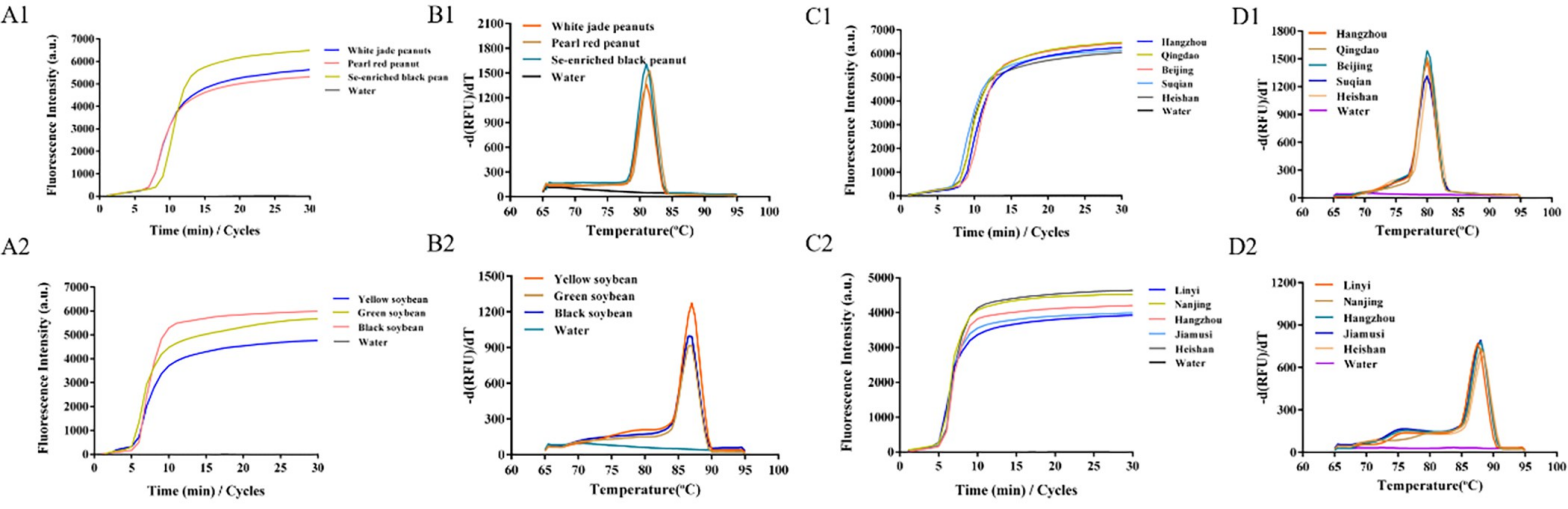
Detection of peanut and soybean allergens in different areas and varieties by LAMP with paper-based microfluidics extraction. Notes: 8A1 & 8C1, amplification curves appeared in peanut samples from different regions and varieties; 8B1 & 8D1, Tm values of peanut from different varieties and different places; 8A2 & 8C2, amplification curves appeared in soybean samples from different regions and varieties; 8B2 & 8D2, Tm values of soybean from different varieties and different places.

Peanut and soybean samples from different areas and varieties were extracted using a paper chip and amplified using the LAMP method to verify the detection of corresponding allergens. Additionally, melting curve analysis was performed for the amplification results. As is shown in **Fig 8**, amplification curves appeared in peanut samples from different regions and varieties (**Fig 8A1 and 8C1**), indicating that the paper chip method can be used for the sample extraction and detection of different varieties and origins. The melting curve showed that the Tm values of peanut samples from different varieties (**Fig 8B1**) and different origins (**Fig 8D1**) were highly consistent, indicating that the amplification templates were all from the DNA extracted on the paper chip. The same results were obtained for soybean samples from different varieties (**Fig 8A2 and 8B2**) and different producing areas (**Fig 8C2 and 8D2**). The aforementioned results suggest that the LAMP detection method of peanut and soybean allergens extracted by paper chip can detect specific sequences of peanuts or soybeans, and the detection results are not affected by origin or variety.

**Fig 9:**
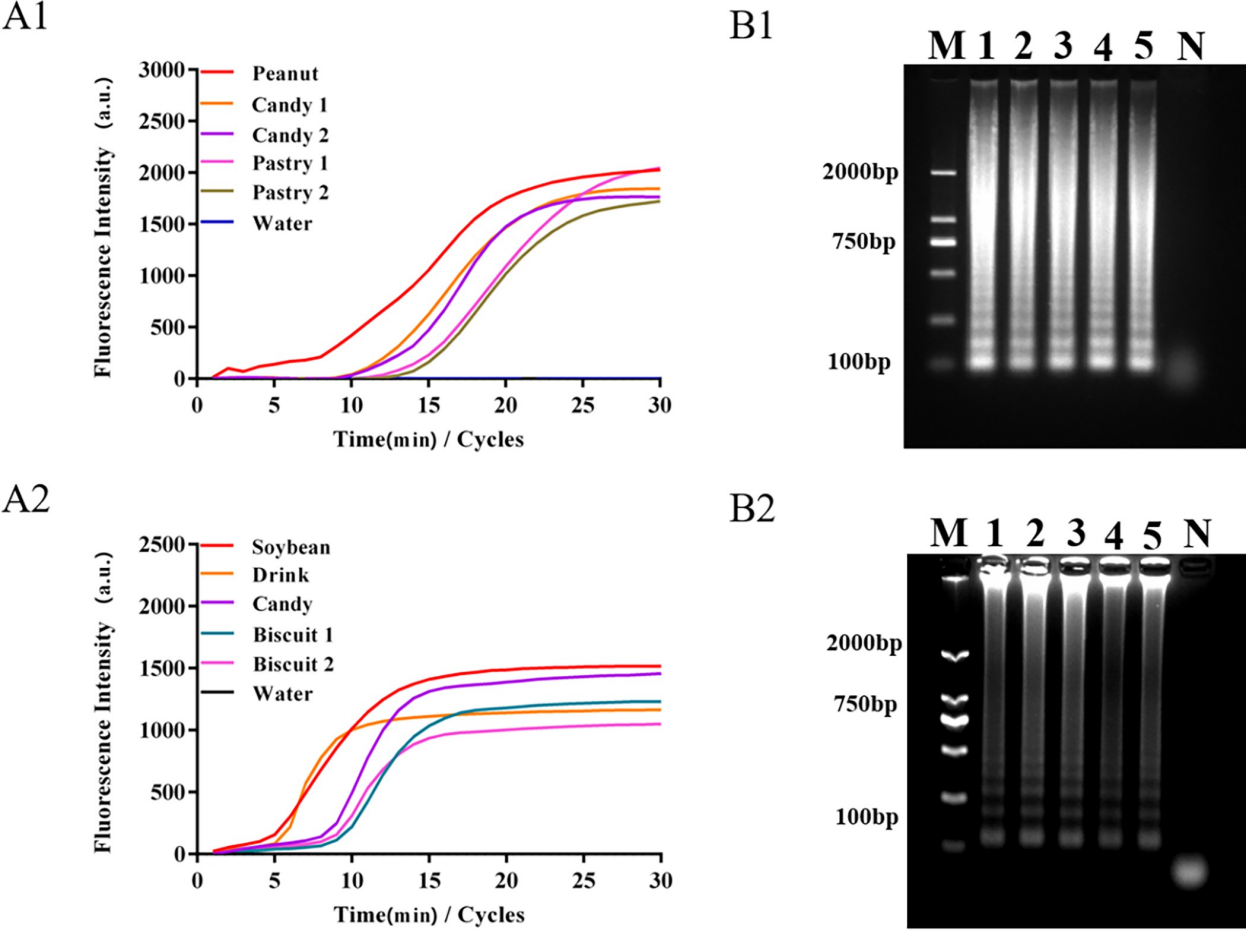
Detection of peanut and soybean allergens in commercial products. Notes: A1 & A2, LAMP amplification curves of different products containing peanut and soybean; B1 & B2, corresponding ladder electrophoresis bands o of different products containing peanut and soybean.

In order to verify the extraction and detection effect of this method on commercial products, eight kinds of products containing peanuts or soybeans (two kinds of candy and two kinds of cakes containing peanuts, and two kinds of drinks and candy as well as two kinds of biscuits containing soybeans) were extracted using a paper chip and amplified by the LAMP method. As is shown in **Fig 9A1 and 9B1**, the LAMP detection method for peanut and soybean allergens extracted by paper chip has a good amplification curve for four commercial commodities containing peanuts. The agarose gel electrophoresis results further verify the detection results of the products sold on the market. As is shown in **Fig 9A2 and 9B2**, the four commercial products containing soybeans also showed good amplification. Subsequently, the corresponding DNA concentrations were calculated based on the amplified Ct values of the eight commercially available products and the linear curves shown in **Fig 5B1 and 5B2** (and the Ct values and DNA concentration **were shown in Table 2**). The aforementioned results show that the method based on paper chip nucleic acid extraction and LAMP detection can achieve the qualitative and quantitative detection of commercial products.

**Table 2:** The detected Ct values and DNA concentration values of commercially available products in this method.

	Sample	Ct value	DNA concentration (ng/μL)
Samples containing peanuts	Positive control	6.54	41.51
	Candy 1	10.02	21.08
	Candy 2	9.97	21.05
	Pastry 1	13.25	2.11
	Pastry 2	13.43	1.06
Samples containing Soybeans	Positive control	5.04	48.19
	Drink	5.58	45.14
	Candy	8.20	30.33
	Biscuit 1	9.45	23.27
	Biscuit 2	9.03	25.65

## 4. Conclusion

In this study, nucleic acids from plant seed allergen (peanut and soybean) by paper-based microfluidics were successfully extracted, and then amplified in LAMP to realize the fast and sensitive detection. The paper-based microfluidics extraction is simple, economical and environmentally friendly. It does not require time-consuming and labor-intensive large-scale equipment to separate and purify DNA. Combined with LAMP detection, paper-based microfluidics extraction can be used in laboratories and POCs. the results show that the method not only has good specificity, sensitivity and repeatability, but also has been successfully applied in the extraction and detection of commercial products. Moreover, this method will support the establishment of an integrated nucleic acid detection method (containing the preparation procedures, extraction and LAMP analysis), which performed on a paper. In conclusion, the established plant seed allergen extraction technique provides reference and support for the development of the quality control of products containing allergens.

## Supporting information

S1 Raw images (PDF)

S1 File (DOCX)
