# Supplementary material for: Rapid identification and quantitation of single plant seed allergen using paper-based microfluidics
Source: PLoS One. 2022 Dec 12;17(12):e0266775. doi: 10.1371/journal.pone.0266775 (PMC9744315; doi:10.1371/journal.pone.0266775)
Supplement: S1 Raw images — (PDF) [file pone.0266775.s001.pdf]

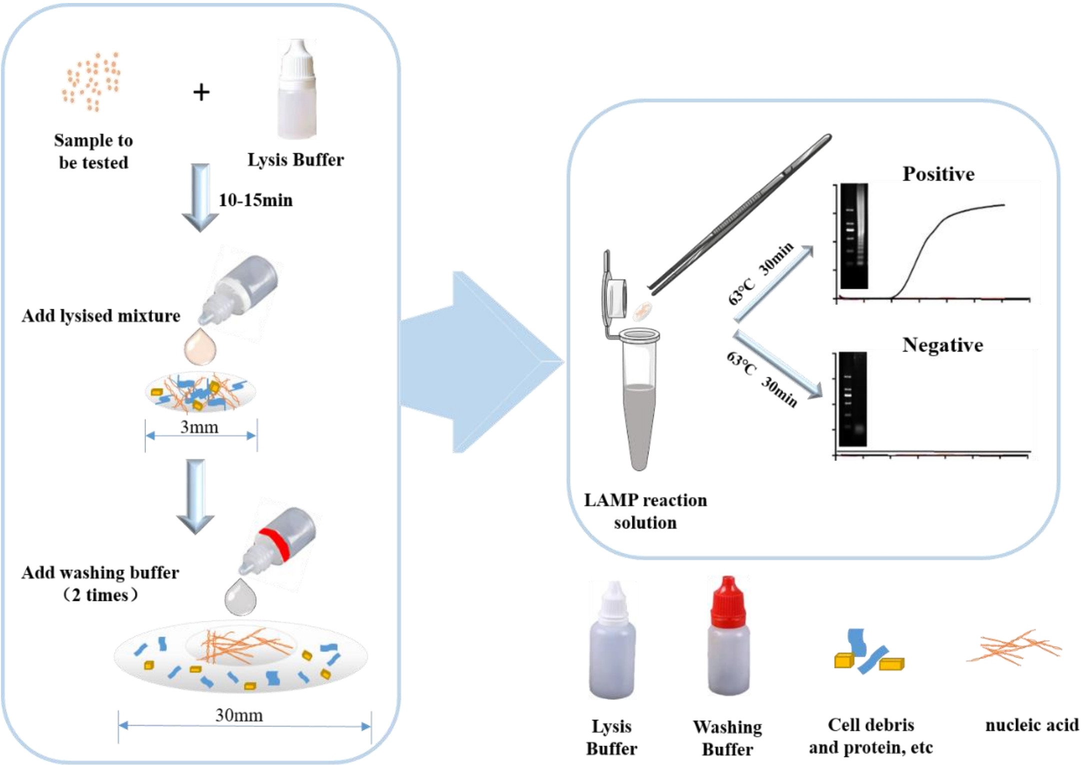

Figure 1 Process of LAMP method for nucleic acid detection extracted by paper-based microfluidics

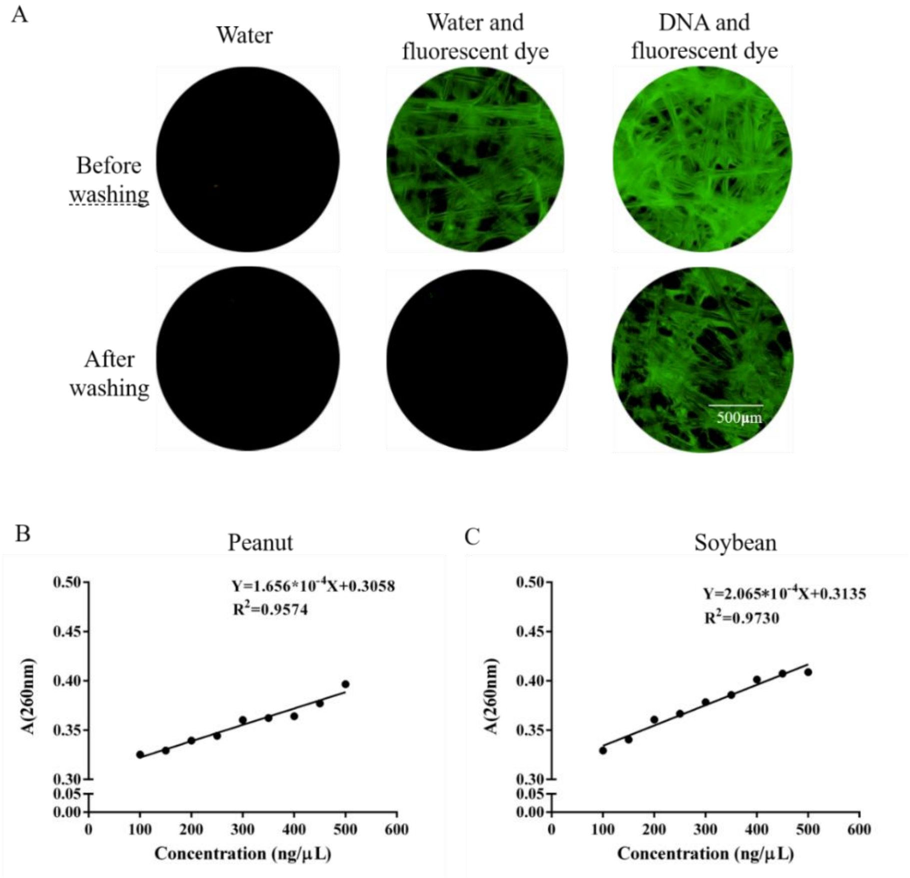

Figure 2 Adsorption of DNA and H_2O on paper-based microfluidics

Notes: A, fluorescence of paper chip; B, standard line of different concentration of DNA.

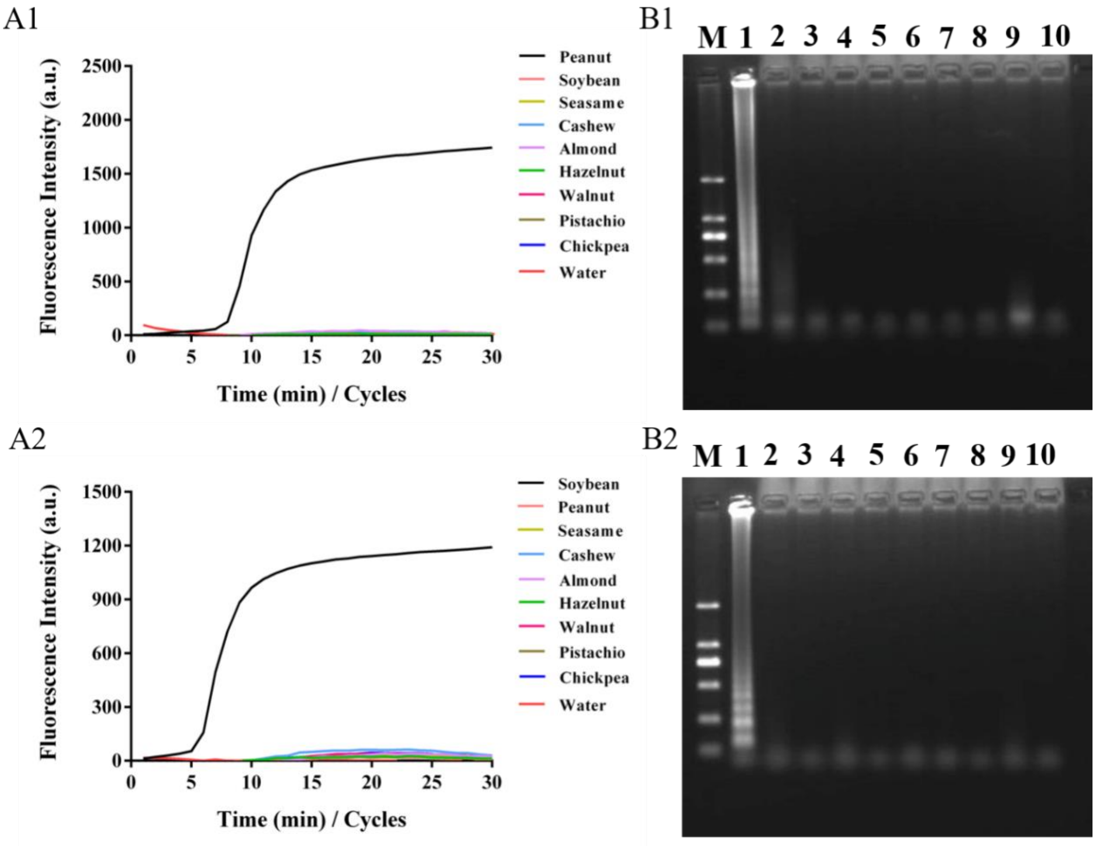

Figure 3 Specificity of LAMP detection with paper-based microfluidics extraction

Notes: A1 & A2, LAMP amplification of peanut and soybean; B1 & B2, corresponding ladder electrophoresis bands of peanut and soybean.

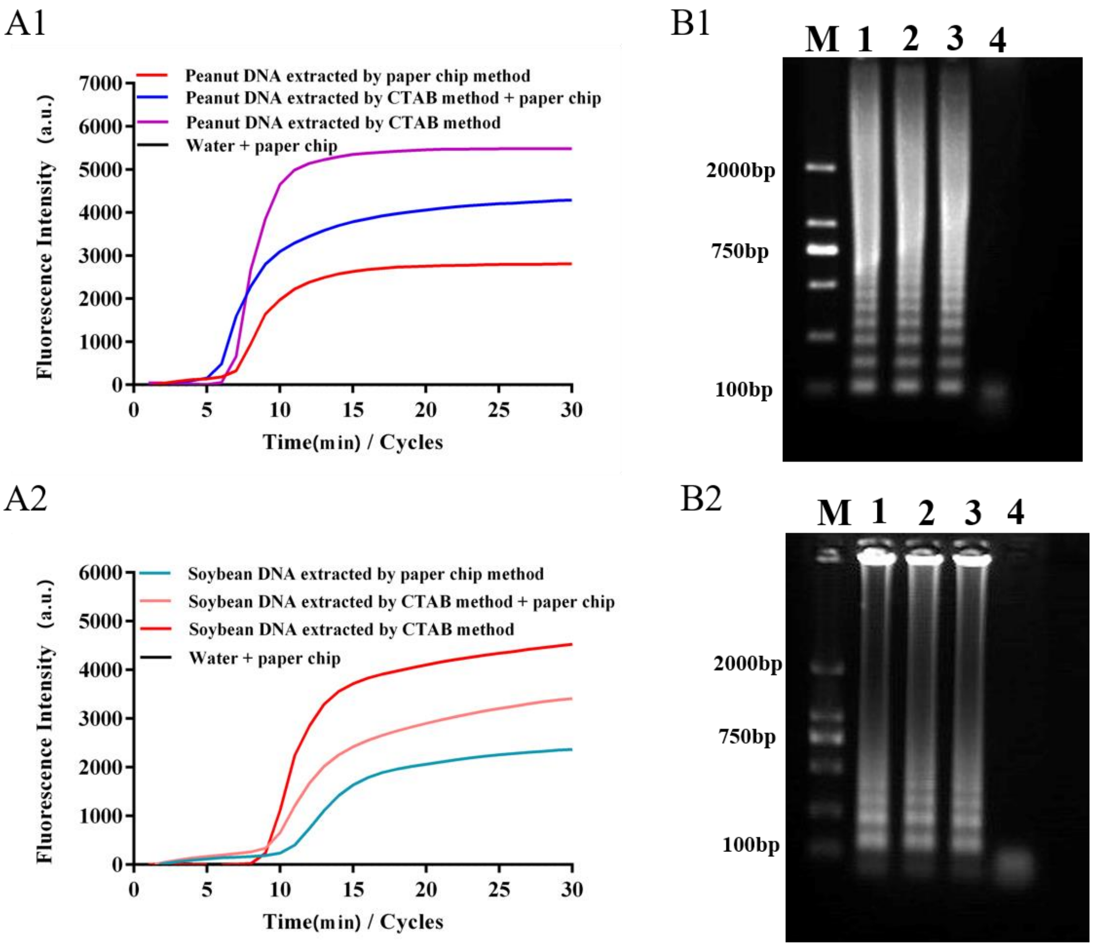

Figure 4 LAMP detection and electrophoresis verification based on the extraction DNA by paper-based microfluidics.

Notes: A1 & A2, LAMP amplification curves of peanut and soybean; B1 & B2, corresponding ladder electrophoresis bands of peanut and soybean.

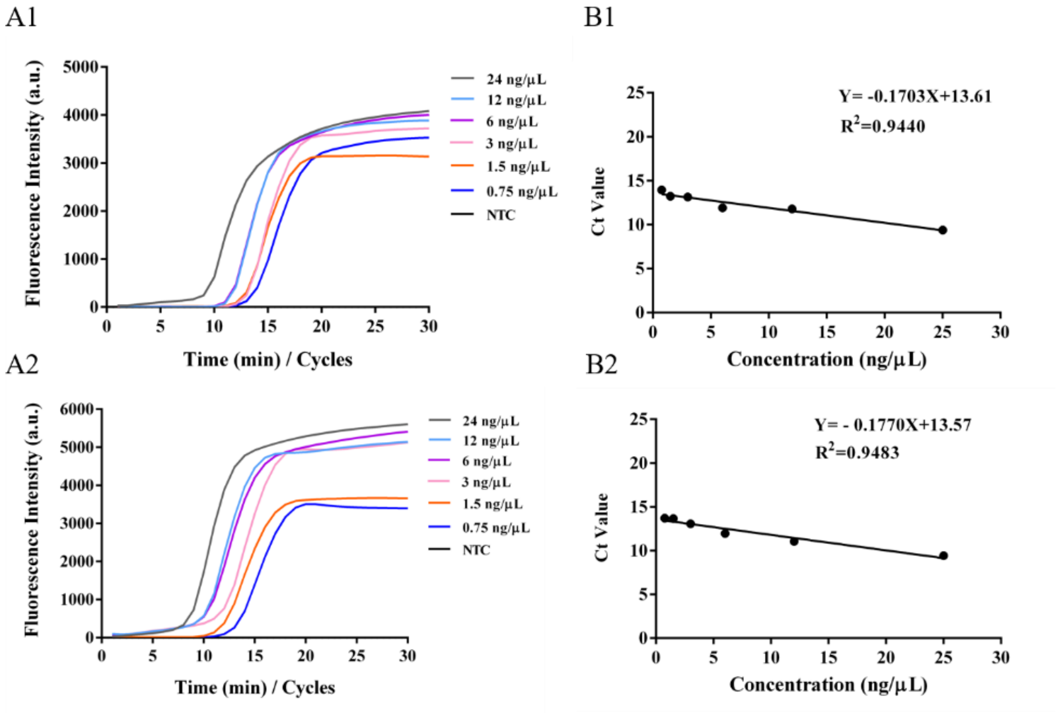

Figure 5 LAMP detection of different concentrations of DNA on paper-based microfluidics

Notes: A1 & A2, amplification curves of peanut and soybean DNA with different concentrations; B1 & B2, linear relationship between peanut and soybean DNA concentrations and Ct value.

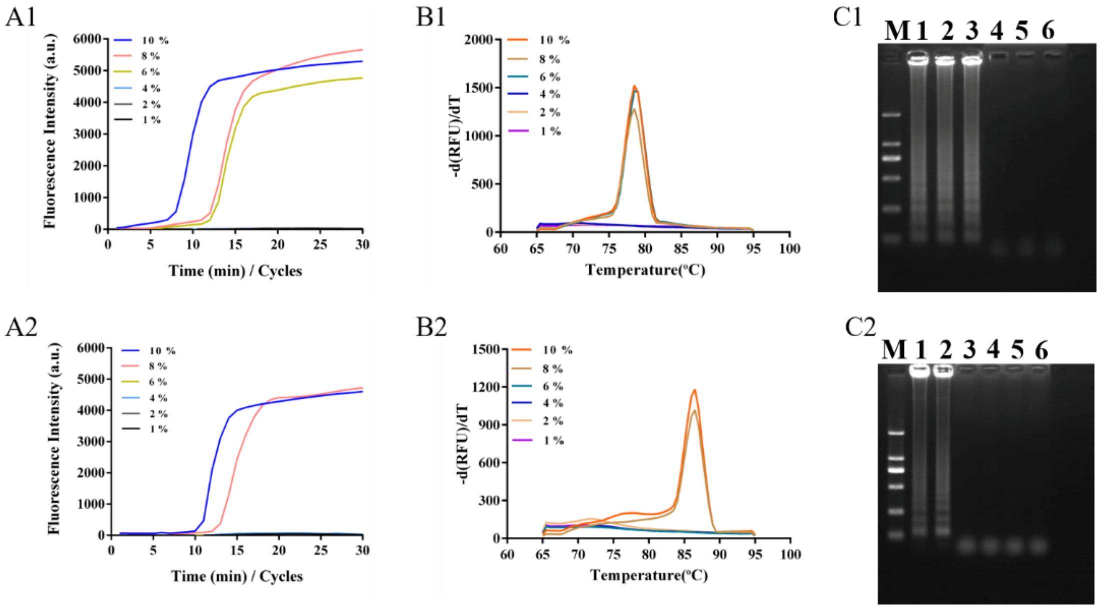

Figure 6 Sensitivity of LAMP detection with paper-based microfluidics extraction. Notes: A1, B1 & C1, the fluorescence amplification results, melting curve results, and agarose gel electrophoresis results of different peanut diluted samples; A2, B2 & C2, the fluorescence amplification results, melting curve results, and agarose gel electrophoresis results of different soybean diluted samples.

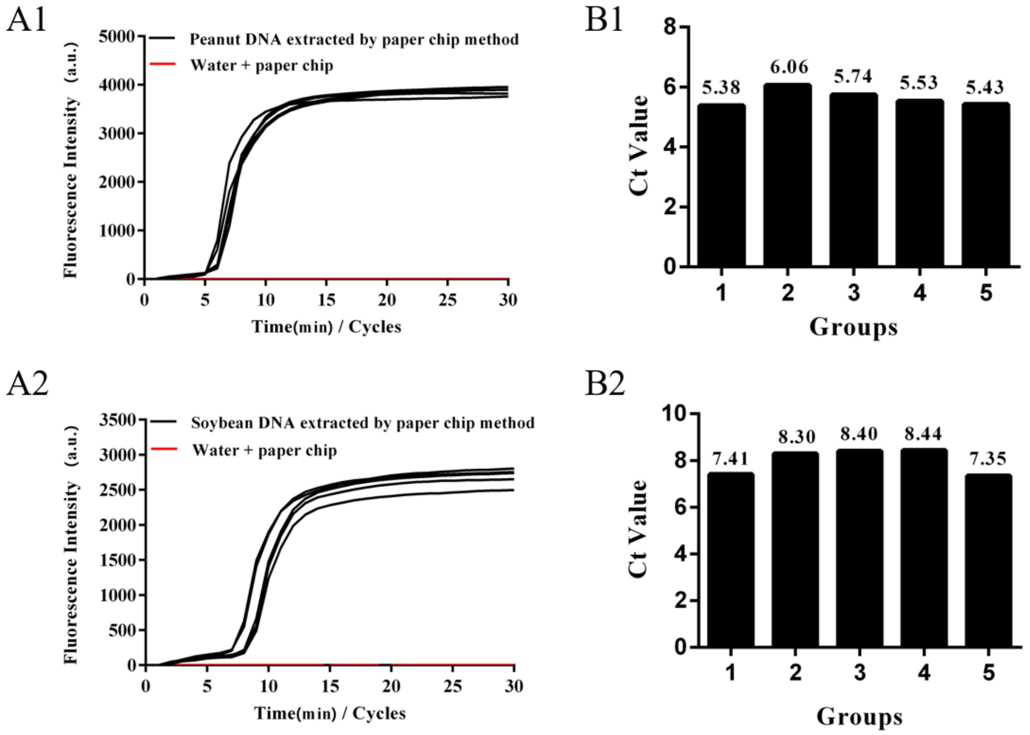

Figure 7 Repeatability of LAMP detection with paper-based microfluidics extraction

Notes: A1 & A2, the amplification results of peanut and soybean with 5 repeats; B1 & B2, Ct value of peanut and soybean with 5 repeats.

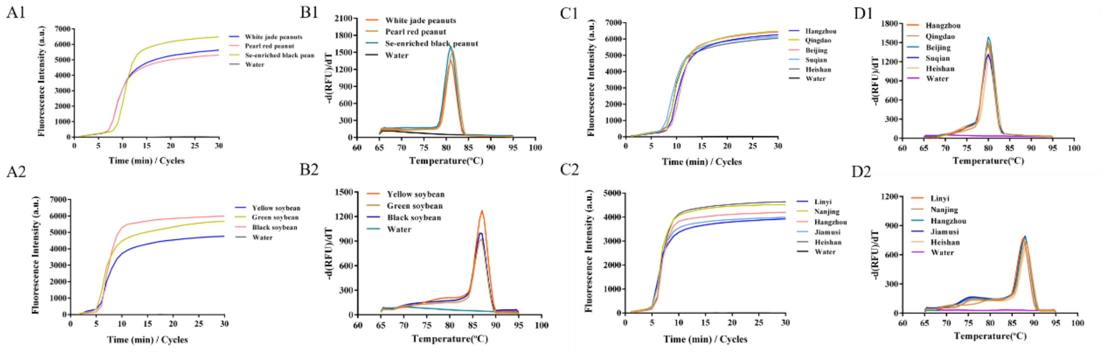

Figure 8 Detection of peanut and soybean allergens in different areas and varieties by LAMP with paper-based microfluidics extraction

Notes: 8A1 & 8C1, amplification curves appeared in peanut samples from different regions and varieties; 8B1 & 8D1, Tm values of peanut from different varieties and different places; 8A2 & 8C2, amplification curves appeared in soybean samples from different regions and varieties; 8B2 & 8D2, Tm values of soybean from different varieties and different places.

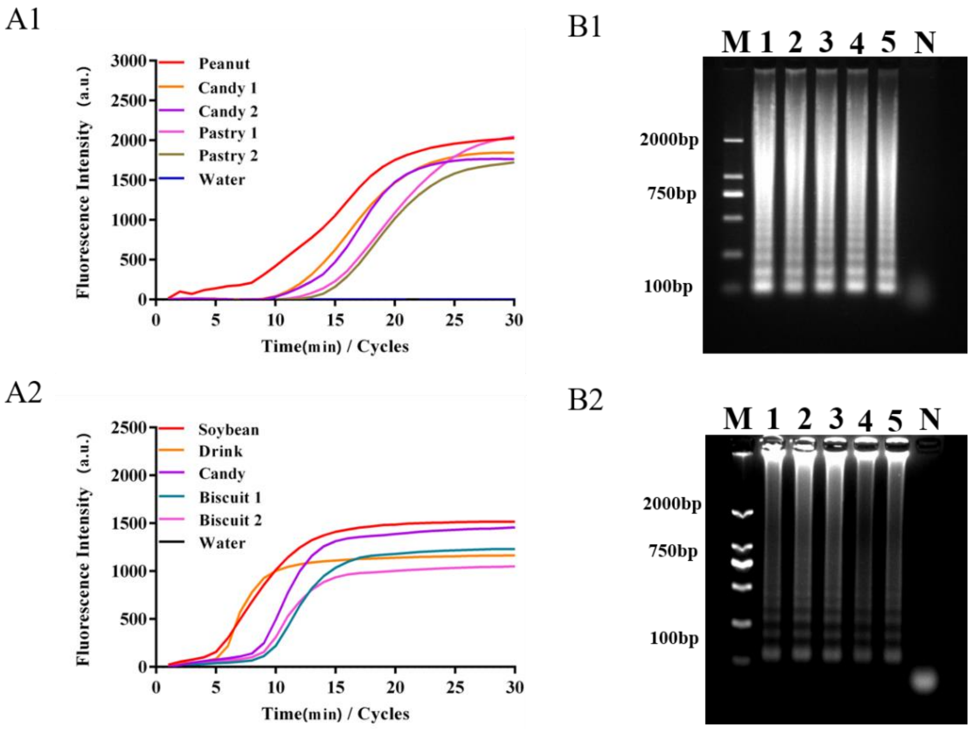

Figure 9 Detection of peanut and soybean allergens in commercial products.

Notes: A1 & A2, LAMP amplification curves of different products containing peanut and soybean; B1 & B2, corresponding ladder electrophoresis bands o of different products containing peanut and soybean.

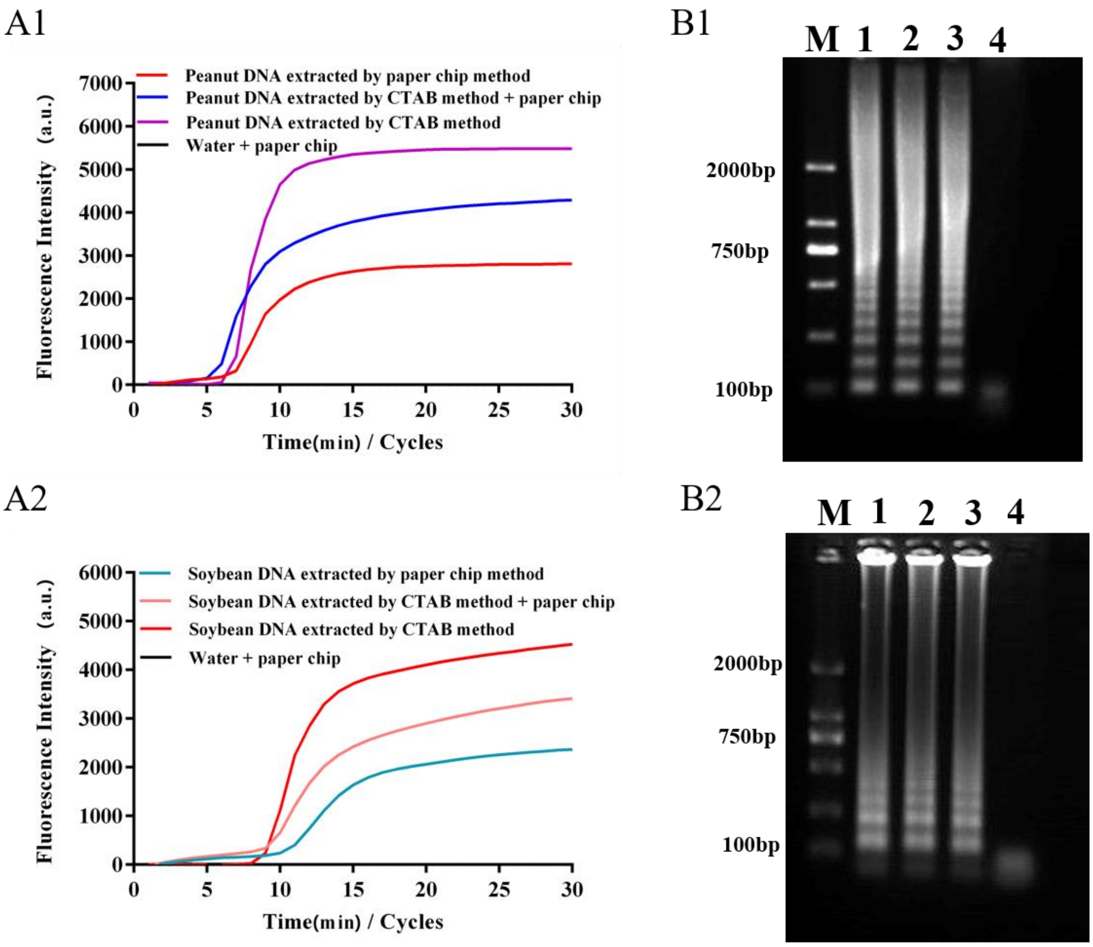

Figure S1 LAMP detection and electrophoresis verification based on the extraction DNA by paper-based microfluidics.
