# Supplementary material for: Rapid identification and quantitation of single plant seed allergen using paper-based microfluidics
Source: PLoS One. 2022 Dec 12;17(12):e0266775. doi: 10.1371/journal.pone.0266775 (PMC9744315; doi:10.1371/journal.pone.0266775)
Supplement: S1 File — (DOCX) [file pone.0266775.s002.docx]

**Supporting Information**

**Rapid identification and quantitation of single plant seed allergen using paper-based microfluidics**

**Xiaodong Sun^b, †^, Yongxin Liu^a, †^, Bing Niu^a^, Qin Chen^a,^** ***, Xueen Fang^c,^** *

^†^ Contribute equally to this work.

^a^ Shanghai Key Laboratory of Bio-Energy Crops, School of Life Sciences, Shanghai University, Shanghai, 200444, P.R. China;

^b^ School of Medicine, Shanghai University, Shanghai 200444, P.R. China;

^c^ Department of Chemistry and Institutes of Biomedical Sciences, Fudan University, Shanghai, 200433, P.R. China;

^*^ Corresponding author at: Shanghai Key Laboratory of Bio-Energy Crops, School of Life Sciences, Shanghai University, Shanghai 200444, P. R. China. E-mail: chenqincc@shu.edu.cn (Qin Chen)

Tel./fax: +86-21-66136276

^*^ Corresponding author at: Department of Chemistry and Institutes of Biomedical Sciences, Fudan University, P. R. China. E-mail: fxech@fudan.edu.cn (Xueen Fang)

**Feasibility of paper microfluidics for the nucleic acids extraction/detection for peanut and soybean allergens**

In order to verify the feasibility of peanut and soybean DNA extracted by paper chip for allergen detection, LAMP detection method was used to detect peanut or soybean DNA extracted by paper chip. The pure peanut or soybean DNA extracted by the CTAB method, the pure DNA and paper chip combined sample and ultrapure water are used as negative control and blank control, and then added to the LAMP system for amplification, and the amplicon was analyzed by agarose gel electrophoresis. The results are shown in **Figure S1**. The fluorescence curve amplification results show that the peanut DNA and soybean DNA extracted by paper chip have very good amplification as other positive samples, the plateau phase was reached within 30 minutes, with high amplification efficiency. And the ladder-like electrophoresis bands in the gel electrophoresis results further verified that the feasibility of our paper chip nucleic acid extraction and LAMP amplification assay for the detection of allergen.


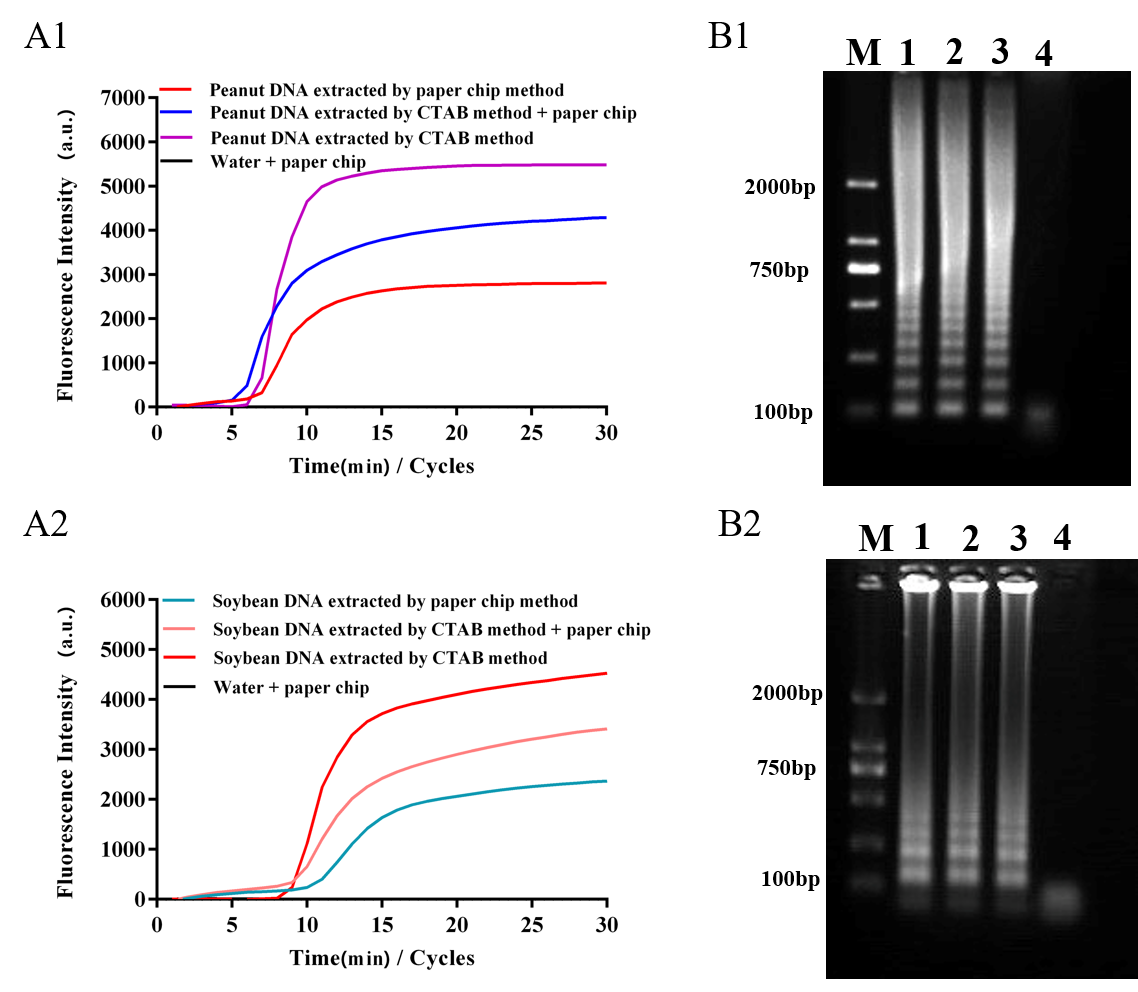


**Figure S1** LAMP detection and electrophoresis verification of peanut (A1&B1) and poybean (A2&B2) based on the extraction DNA by paper-based microfluidics.

**Table S1** A comparison of the present methods with some other works

| **Extraction methods** | **Detection methods** | **Merits** | **Samples** | **Reference** |
| --- | --- | --- | --- | --- |
| magnetic nanoparticles | PCR | automatically | Listeria monocytogenes nucleic acid | [1] |
| paper-based device | helicase-dependent isothermal amplification  and LFA detection | integration, simple | Salmonella Typhimurium nucleic acid | [2] |
| paper microfluidic | PCR | Rapid, Smartphone-based detection | Salmonella Typhimurium nucleic acid | [3] |
| paper disc | LAMP | Flexible, High throughput | nucleic acid of rotavirus A | [4] |
| **paper-based microfluidics** | **LAMP** | **Fast, Flexible, Low cost** | **peanut and soybean** **nucleic acid** | **This work** |

[1] Yu Fu, Xiaoming Zhou, Da Xing. Integrated paper-based detection chip with nucleic acid extractionand amplification for automatic and sensitive pathogen detection. Sensors and Actuators B: Chemical, 2018, 261, 288-296.

[2] Christopher F. Fronczek, Tu San Park, Dustin K. Harshman, et al. Paper microfluidic extraction and direct smartphone-based identification of pathogenic nucleic acids from field and clinical samples. RSC Adv., 2014,4, 11103-11110.

[3] Ruihua Tang, Hui Yang, Yan Gong, et al. A fully disposable and integrated paper-based device for nucleic acid extraction, amplification and detection. Lab Chip, 2017,17, 1270-1279.

[4] Xin Ye, Jin Xu, Lijuan Lu, et al. Equipment-free nucleic acid extraction and amplification on a simple paper disc for point-of-care diagnosis of rotavirus A, Analytica Chimica Acta, 2018, 1018, 78-85.
